# Preclinical evaluation of endoscopic placement of a steroid-eluting metal stent in an in vivo porcine benign biliary stricture model

**DOI:** 10.1038/s41598-022-12957-0

**Published:** 2022-05-25

**Authors:** Sung Ill Jang, Sungsoon Fang, Ji Hae Nahm, Jae Hee Cho, Min Young Do, Su Yeon Lee, Seok Jeong, Don Haeng Lee, Dong Ki Lee

**Affiliations:** 1 Department of Internal Medicine, Gangnam Severance Hospital, Yonsei University College of Medicine, 211 Eonjuro, Gangnam-gu, Seoul, 06273 Republic of Korea; 2 Severance Biomedical Science Institute, BK21 Plus Project for Medical Science, Gangnam Severance Hospital, Yonsei University College of Medicine, Seoul, Republic of Korea; 3 Department of Pathology, Gangnam Severance Hospital, Yonsei University College of Medicine, Seoul, Republic of Korea; 4 Department of Internal Medicine, Inha University Hospital, Inha University College of Medicine, Incheon, South Korea; 5 Utah-Inha DDS & Advanced Therapeutics Research Center, Incheon, Republic of Korea

**Keywords:** Diseases, Gastroenterology

## Abstract

Treatment of benign biliary strictures (BBS) using fully covered self-expandable metal stents (FCSEMS) has a high success rate, but recurrence can occur. Corticosteroids may be useful based on their anti-fibrotic and anti-inflammatory effects. We investigated the safety and efficacy of corticosteroid-eluting FCSEMS in an animal model. BBSs were created by radiofrequency ablation in 12 mini-pigs. Four weeks later, FCSEMS coated with 0 mg (control), 15 mg (steroid 1 × group), or 30 mg (steroid 2 × group) triamcinolone were inserted endoscopically. The in vitro drug release assay revealed that the optimal stent had 15 mg of triamcinolone and a hydrophilic membrane. No transmural necrosis or perforation occurred in any animal. Fibrous wall thickness tended to decrease macroscopically and microscopically in a triamcinolone dose-dependent manner (control *vs*. steroid 2 × group: 773.1 *vs*. 468.5 µm, P = 0.037). Thickness also decreased over time in the steroid 2 × group (3 days *vs*. 4 weeks: 907.9 *vs*. 468.5 µm, P = 0.025). Blood parameters tended to improve after stent insertion. In a porcine BBS model, steroid-eluting FCSEMS showed potential as a safe and effective treatment modality to reduce fibrotic tissue. Studies are required to confirm their safety and efficacy in humans with refractory BBS.

## Introduction

Benign biliary strictures (BBS) have a number of antecedents, including postoperative stenosis, chronic pancreatitis, gallstones, primary sclerotic cholangitis, ischemia, autoimmune pancreatitis, and radiation therapy. Among these, postoperative stenosis and chronic pancreatitis are the most frequent^1^. Biliary stent implantation using endoscopic retrograde cholangiopancreatography (ERCP) is the preferred treatment for BBS^2–4^.

Both plastic stents and self-expanding metal stents are available for biliary tract implantation. Plastic stent implantation has a success rate of 80–90% for benign stenosis after liver transplantation and 50–60% for benign stenosis caused by chronic pancreatitis^3,5,6^. Although plastic stent implantation is usually performed first because it has fewer complications, many ERCP procedures must be performed to obtain therapeutic effects.

Fully covered self-expandable metal stents (FCSEMS) have been developed to overcome the shortcomings of plastic stents. Because of their larger diameter and self-expanding nature, FCSEMS are a highly effective treatment for BBS, and one or two fewer ERCPs are required to achieve therapeutic benefits with FCSEMS than with plastic stents^7,8^. However, FCSEMS are associated with a 16% rate of stricture recurrence and the possibility of stent migration from the biliary tract^9–11^.

In BBS, recurrent stenosis after plastic or metal stent insertion is at least partly caused by elastic recoil of the biliary tract. Restenosis may also result from transmural fibrosis caused by inflammation developing as a consequence of the mucosal tearing that occurs with stenosis expansion^12–14^. Inflammation in the biliary tract can lead to marked fibroblast proliferation, excessive synthesis and deposition of collagen due to large-scale aggregation of macrophages, and synthesis and secretion of polypeptide growth factors. All of these effects can lead to bile duct stenosis^14^. Steroid injection or oral steroid medication can be used to prevent esophageal stricture after endoscopic dissection and BBS^15–20^. Therefore, an anti-fibrotic agent, such as a corticosteroid, can be used to prevent restenosis of the biliary tract secondary to fibrosis.

We investigated the safety and efficacy of a corticosteroid-eluting FCSEMS in a porcine model of BBS induced by radiofrequency ablation (RFA).

## Results

### In vitro drug release

**Figure 1:**
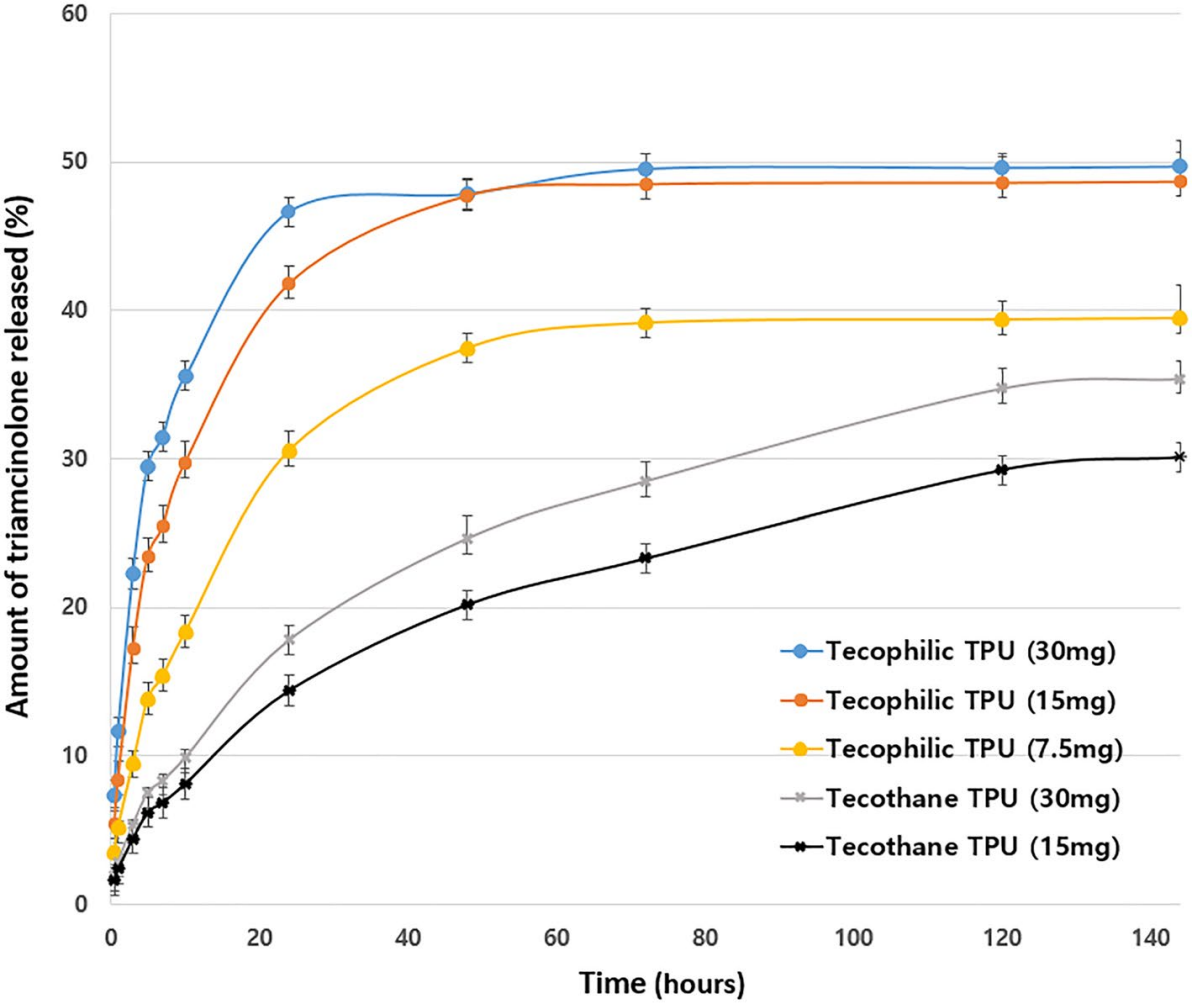
In vitro drug release profile. In vitro cumulative release of triamcinolone over the first 6 days, measured by high-performance liquid chromatography, from experimental stents with various concentrations of triamcinolone and types of polyurethane.

The mean rate of drug release from stents coated with the two types of hydrophobic polyurethanes was ~ 13% lower than that from stents coated with the three types of hydrophilic polyurethane after 144 h (Fig. 1). Furthermore, drug release from the hydrophilic polyurethane stents was maintained for 6 days.

An initial burst of > 30% triamcinolone was observed within the first day of the experiment from all of the triamcinolone hydrophilic polyurethane stents, whereas no burst of triamcinolone release occurred within the first day from the hydrophobic polyurethane stents. At 3 days, the amount of steroid released from the 15 and 30 mg triamcinolone stents was similar, and significantly greater than the amount released from the 7.5 mg triamcinolone stent (30 mg vs. 15 mg, P = 0.612; 15 mg vs. 7.5 mg, P = 0.006).

At 6 days, the cumulative percentage of drug released from the 15 and 30 mg triamcinolone hydrophilic polyurethane stents was approximately 50%. By contrast, the cumulative percentages of drug released from the 7.5 mg triamcinolone hydrophilic stent, 30 mg triamcinolone hydrophobic stent, and 15 mg triamcinolone hydrophobic stent were notably lower (40% and 35.7%, respectively). Taken together, our results indicate that the 15 mg triamcinolone hydrophilic polyurethane stent exhibited release behavior similar to the 30 mg triamcinolone hydrophilic polyurethane stent but the absolute amount of steroid released from the 15 mg triamcinolone hydrophilic polyurethane stent was lower than that released from the 30 mg triamcinolone hydrophilic polyurethane stent. Thus, the 15 mg hydrophilic polyurethane stent was considered as optimal concentration for safety.

### Outcome following RFA and stent insertion

One animal died 3 days after RFA. The autopsy showed duodenal perforation, which likely occurred during the ERCP procedure. The death was unrelated to the RFA procedure because the extracted bile duct exhibited no perforation or necrosis. Body weight was measured once per week during the experiment. No significant difference in body weight was observed between the groups before or after inserting the stents, suggesting that the drug-releasing stent had no major systemic side effects. No stent migrated in any animal during the experiment.

### Historical changes

**Figure 2:**
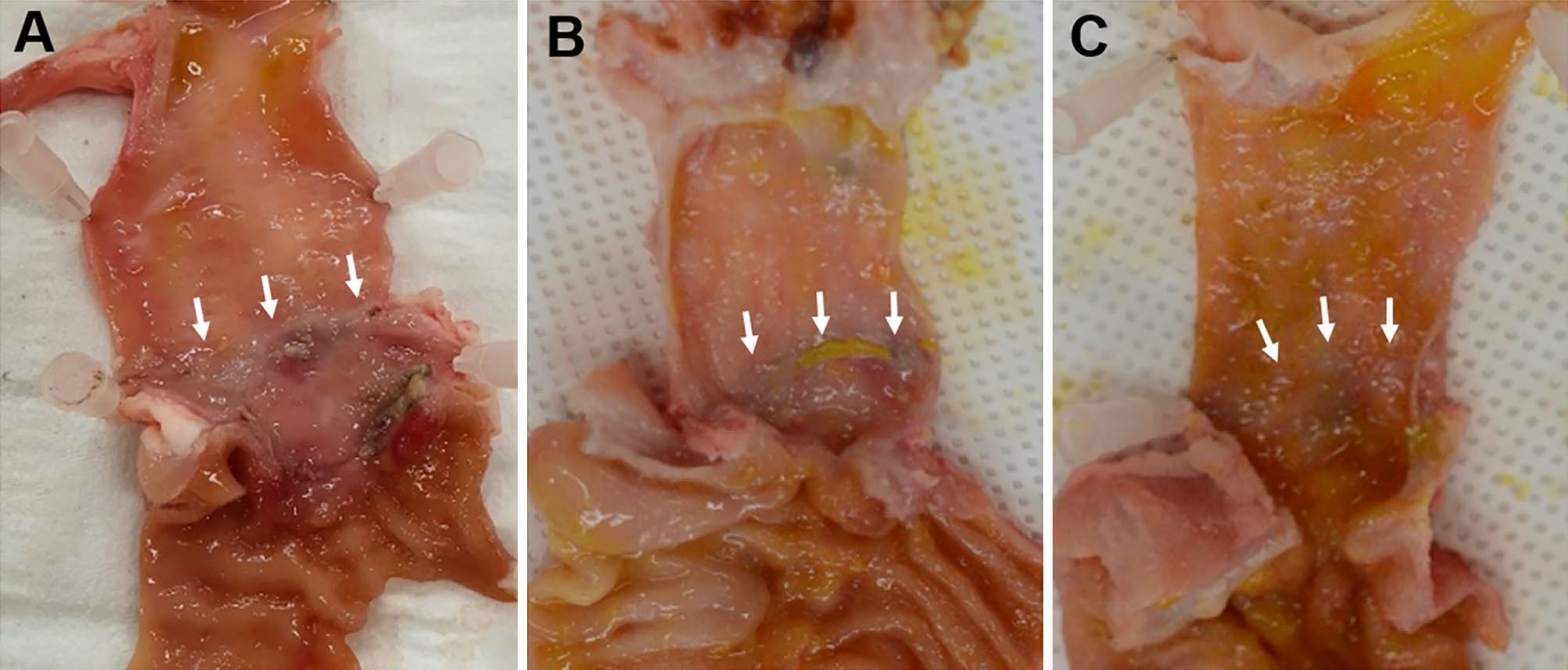
Gross pathology. Gross pathology at 4 weeks after stent insertion showing better healing in the stricture area (white arrow) in the steroid-eluting stent groups (**B**, 15 mg triamcinolone; **C**, 30 mg triamcinolone) compared to the control group (**A**). The degree of healing was greatest in **C**, reflecting a dose-dependent relationship between the extent of healing and steroid dose.

At 4 weeks after stent insertion, gross examination of the CBDs revealed a greater effect of treatment at the stenosis site in the steroid-eluting stent groups than the control group (Fig. 2). The effect of treatment was markedly greater in the steroid 2 × (30 mg triamcinolone) group. No perforation or necrosis was observed at the stent area in any animal, thereby confirming the safety of the steroid-eluting stents.

**Figure 3:**
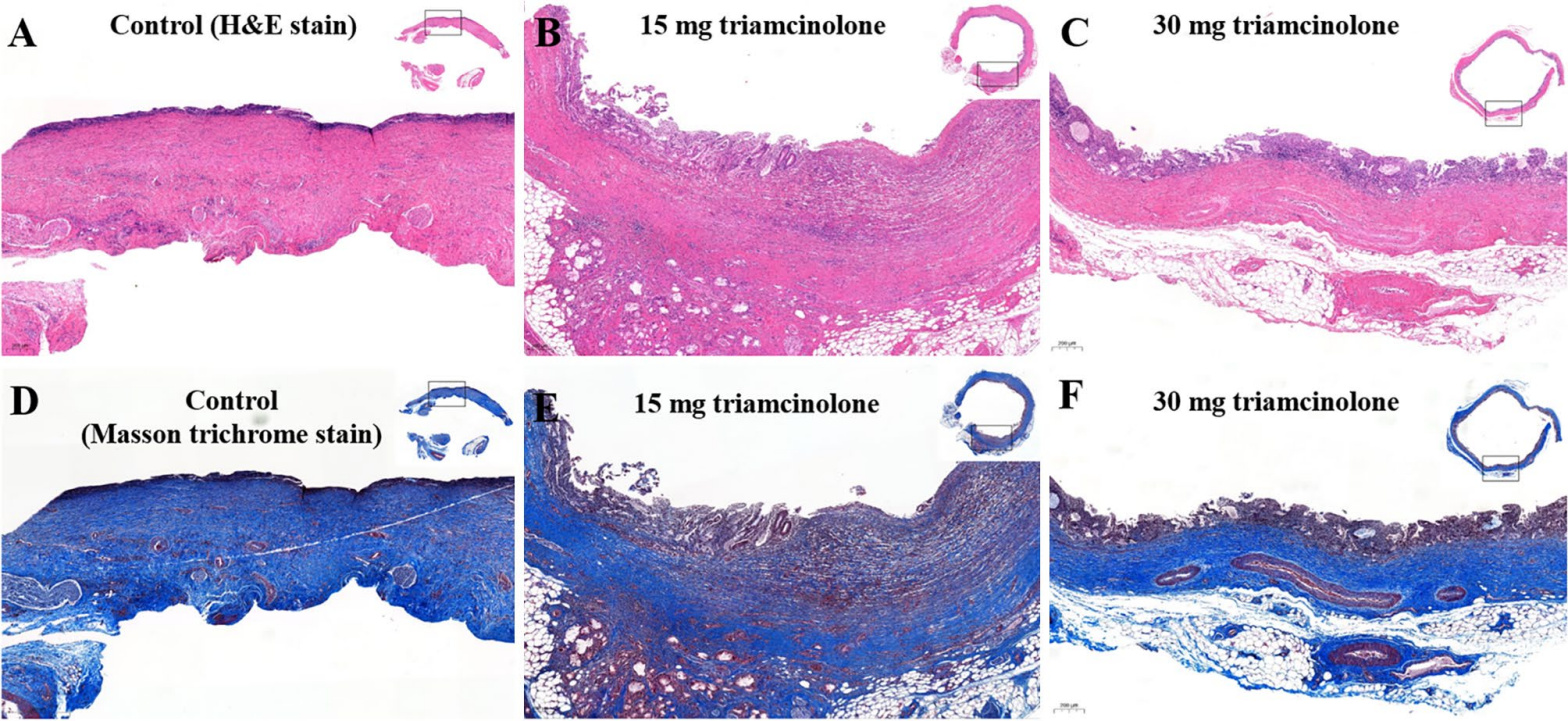
Microscopic pathology. Microscopic appearance of H&E-stained and Masson trichrome-stained sections of the bile duct 4 weeks after stent insertion. Control group (**A**) showing eroded epithelium, thick fibrosis, and moderate inflammation. Triamcinolone (15 mg) steroid-eluting stent group (**B**) showing residual erosion with regenerated epithelium, moderate inflammation, and partially resolved fibrosis. Triamcinolone (30 mg) steroid-eluting stent group (**C**) showing complete epithelial recovery, mild to moderate inflammation, and less fibrosis than the other groups.

**Figure 4:**
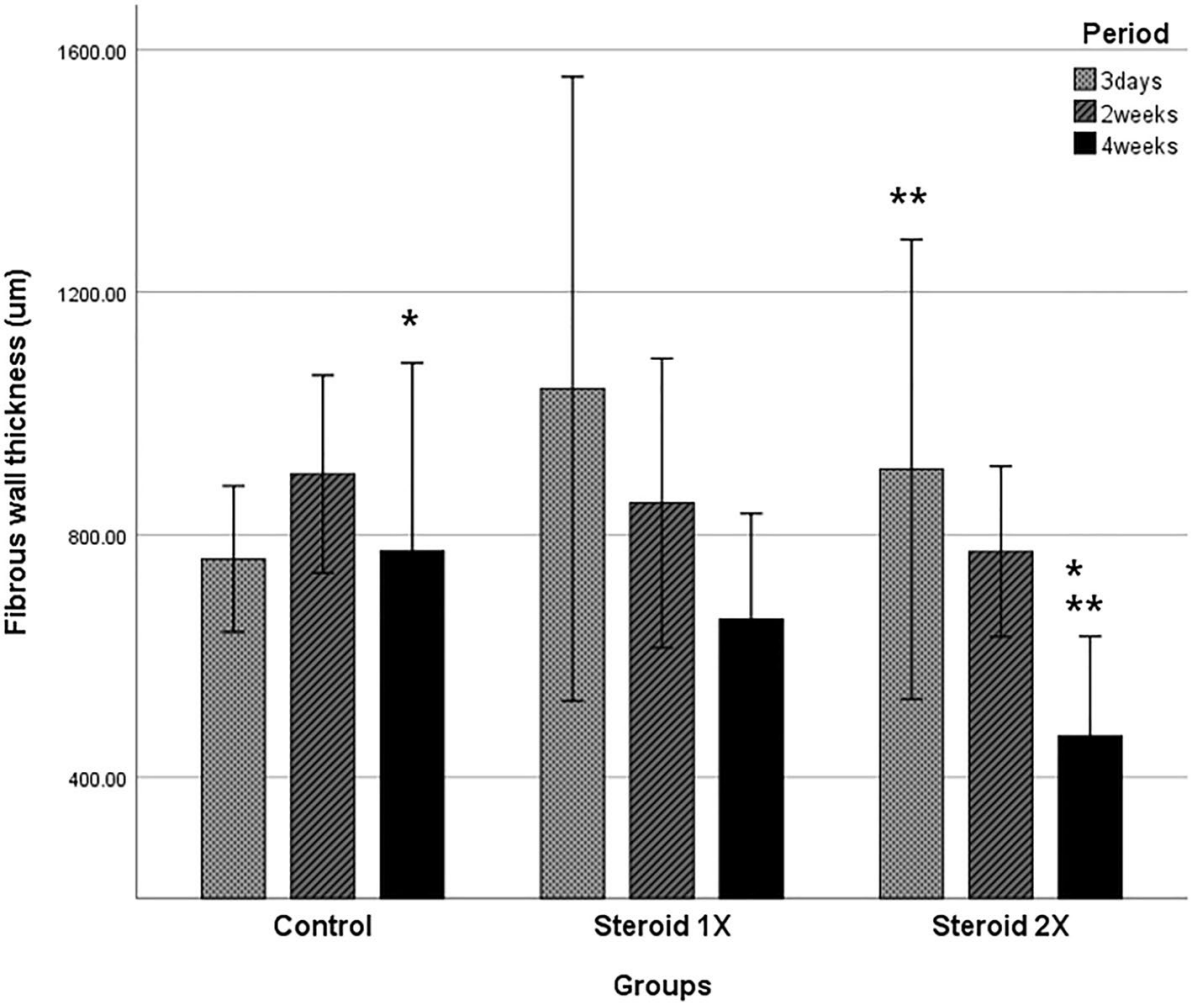
Fibrous wall thickness. There was no change in fibrous wall thickness in the control group over time, whereas fibrous wall thickness tended to decrease over time in the steroid-eluting stent groups. When comparing fibrous wall thickness before and 4 weeks after steroid-eluting stent insertion, the decrease was statistically significant in the steroid 2× (30 mg triamcinolone) group, but not in the steroid 1x (15 mg triamcinolone) group. *Comparison of fibrous wall thickness between the control and steroid 2 × groups at 4 weeks after steroid-eluting stent insertion (773.1 vs. 468.5 µm, P = 0.037). **Comparison of fibrous wall thickness between 3 days and 4 weeks after steroid-eluting stent insertion in the steroid 2 × group. (907.9 vs. 468.5 µm, P = 0.025).

On histologic examination of the CBDs at 3 days after RFA, all three groups exhibited ulceration with denuded epithelium and necro-inflammatory exudate, along with moderate transmural acute and chronic inflammation, and the deposition of coarse, dense collagenous bundles with edema. Over time, the lining epithelium regenerated, the degree of inflammation decreased, and fibrosis increased. After 4 weeks, the epithelium was mostly replaced by regenerated epithelium, and there was markedly less inflammation and fibrosis in the steroid 2 × group than in the steroid 1 × and control groups (Fig. 3). Fibrous wall thickness tended to decrease in a dose-dependent manner (Fig. 4).

### Blood analyses

CBC and biochemistry blood tests were obtained before RFA, 4 weeks after RFA, and 3 days, 2 weeks, and 4 weeks after stent insertion. In general, the values increased after creation of the stenosis but showed a decreasing trend after stent insertion (Supplementary Fig. 1). There were no statistically significant differences between groups for any test at any time point.

## Discussion

Although endoscopic treatment of BBS with FCSEMS or plastic stents is initially effective^8,13^, existing surgical, endoscopic, and percutaneous treatments for BBS do not guarantee long-term resolution. BBS recurrence has been observed even using methods with high long-term stricture resolution rates^13^. Furthermore, multiple sessions or procedures are required with conventional methods, which is a distinct disadvantage for patients with BBS considering the benign nature of the disease and thus the long life expectancy. Repeated procedures for treating refractory BBS increase medical costs and cause considerable inconvenience to patients. Therefore, new treatment strategies are required. In this animal study, we confirmed that the rate of recurrent stricture in BBS could be reduced by reducing fibrotic wall thickening using steroid-eluting FCSEMS. Safety was also confirmed: no necrosis of the bile duct was observed. The results suggest that steroid-eluting FCSEMS may be effective for minimizing BBS recurrence and thus reducing the need for repeat ERCP procedures for stent insertion.

The pathophysiological mechanisms underlying BBS development include an inflammatory response, collagen deposition and fibrosis formation^12^. Therefore, corticosteroids, with their well-established anti-inflammatory and anti-fibrotic effects, can be administered. Corticosteroids are widely used to prevent fibrosis. Intralesional corticosteroid injections are used in the treatment of postoperative scars, acting through suppression of proinflammatory mediators to reduce fibroblast proliferation, collagen synthesis, and glycosaminoglycan synthesis^14^. Corticosteroid injection of esophageal strictures refractory to conventional treatment can reduce the number of balloon dilations required for treatment, as well as prolong the interval between dilations^21^. For colon strictures secondary to Crohn’s disease, a combination of balloon dilation and corticosteroid injection reduces the frequency of re-expansion procedures or surgery compared with balloon dilation alone^22^. Oral prednisolone, triamcinolone injections, and polyglycolic acid sheets with triamcinolone injections can prevent the occurrence of strictures after esophageal endoscopic dissection^17,23,24^. The combination of triamcinolone injection and shielding with polyglycolic acid sheets and fibrin glue is also effective for preventing and reducing strictures after esophageal endoscopic submucosal dissection^17,25–27^.

Previous studies have confirmed the safety and efficacy of directly injecting corticosteroids into areas of gastrointestinal stenosis. However, these methods lead to an uneven distribution of the corticosteroid and require a high degree of control during injection. In addition, the steroid is not released steadily, which reduces the effectiveness of these methods and decreases their ability to consistently prevent the stricture^17^. Care must also be taken to avoid injection of steroid into the muscularis propria^17^. By contrast, delivering corticosteroids via FCSEMS allows steady release and restricts the steroid to the mucosal layer in direct contact with the stent. In this study, triamcinolone released from steroid-eluting stents diffused evenly over the fibrotic tissue. In addition, only mucosal denudation was observed in the bile duct contacting the stent, and there was no necrosis or perforation.

Safety and efficacy have been confirmed for balloon dilation and corticosteroid injection of biliary-biliary stenosis after liver transplantation, as well as for biliary-gastrointestinal stenosis after pancreatic biliary surgery^18,20^. Although few studies have been conducted, percutaneous transhepatic corticosteroid injection combined with balloon dilation appears to be safe and effective in the long term for BBS, and could be an alternative treatment for these strictures^18^. However, the strategy includes a steroid injection into the stricture site, which can lead to uneven diffusion and stricture enlargement due to balloon dilation, and this to a lack of continuous dilation over a prolonged period. In addition, this method requires the creation of a percutaneous tract and repeated treatments.

Local treatment using drug-eluting stents was first applied to malignant strictures^28,29^. Local anti-tumor effects using paclitaxel-eluting stents have been demonstrated in malignant biliary strictures caused by extrahepatic bile duct cancer. Likewise, steroid-eluting stents are expected to have local anti-fibrotic effects in BBS. Metal stenting in biliary stenosis does not require additional procedures, such as percutaneous transhepatic biliary drainage or biliary duct dilation. In addition, it provides constant release of the corticosteroid loaded in the stent, thus producing uniform effects with a small dose. Therefore, based on this animal study, steroid-eluting FCSEMS are expected to serve as a safe and effective treatment for refractory BBS in humans. It is necessary to apply and evaluate other anti-fibrotic agents besides steroids in drug-eluting stents. Furthermore, techniques to control drug release are needed. A drug coating in the form of a nano-granulated state is needed to prevent initial burst release^30^, and a surfactant, such as Pluronic F-127, can be used for the long-term and continuous release of high concentrations of an anti-fibrotic agent added to the membrane^31^.

In this study, the optimal amount of triamcinolone was 15 mg based on the results of the in vitro drug release test. Drug release was similar after 144 h for the 15 and 30 mg triamcinolone stents. However, in our pig model, the 30 mg triamcinolone stents were more effective in reducing fibrous wall thickening, and the effectiveness was dose- and time-dependent. This superior effectiveness was likely the result of an increase in the amount of triamcinolone released from the 30 mg stent during the 4-week maintenance period. Considering that the amount of triamcinolone used in previous studies of esophageal strictures was 30 mg^17,18,23,25^, the optimal dose may be 30 mg for steroid-eluting FCSEMS. Therefore, this is a limitation of the current study. Additional studies are required to identify the optimal corticosteroid concentration and stent indwelling period, and to assess the long-term drug release pattern.

In this study, RFA was used to create the BBS model. Previous in vivo swine experiments of temperature-controlled EB-RFA reported short- and long-term safety and efficacy of EB-RFA^32,33^. At 4 weeks after EB-RFA, histologic examination revealed a fibrous band at the level of the stricture, and gross examination revealed diffuse, reddish mucosal inflammation of the proximal CBD. The bile duct wall was thickened and composed of mucosal ulcerations, reactive myofibroblastic proliferation, dense collagen deposition, fat necrosis, and inflammatory cell infiltration. However, there were no long-term gross or microscopic adverse events, such as perforation or hemorrhage^33^. Wound healing is inevitably accompanied by varying degrees of cicatrix formation. Deposition of excessive amounts of collagen in lesion regions can lead to over-proliferation of cicatrix^14^. The main manifestations of postoperative BBS are scar contracture and stenosis of the bile duct, but the mechanism underlying stricture formation remains unclear. In a dog model of bile duct trauma repair, the main cause of scar contracture and stricture was myofibroblasts^12^. High expression levels of CD68, TGF-β1, and α-SMA are closely related to fibroblast proliferation, extracellular matrix over-deposition, and scar contracture in bile ducts^12^. Steroids have anti-inflammatory and anti-fibrotic effects, and are expected to reduce fibrotic wall thickening by inhibiting myofibroblasts. Further studies on the mechanism of steroid-eluting FCSEMS are needed.

First limitation of the current study was that drug release was observed for only 144 h, and the follow-up after inserting the stent was 4 weeks, which is short term. In the future, it may be necessary to measure the amount of steroid released over a longer time in a long-term in vitro release test and to study the long-term complications in animal experiments. However, the amount currently loaded in the stent in this study was 30 mg, which is less than the amount used for the steroid injection in previous clinical studies. Furthermore, as the release time of 50% of the total loaded amount is about 1 week, and this animal experiment was conducted for about 4 weeks, no long-term complications were expected. Second limitation was that we were unable to determine whether restenosis occurred after stricture resolution. This is an inherent limitation of most animal studies. As long-term stricture resolution without restenosis after stent removal is the ultimate goal of steroid-eluting stents, long-term investigations in humans are required to explore this issue. Third limitation was that our BBS model involved a stricture formed by inducing inflammation, which does not reflect all types of BBS. Clinical studies are required to explore the use of steroid-eluting stents in humans with BBS of various etiologies.

In conclusion, the safety and efficacy of steroid-eluting stents was confirmed in an animal model of BBS induced by EB-RFA. Steroid-eluting stents can exert a mechanical effect that dilates the CBD, and a chemical effect that reduces fibrosis via anti-inflammatory and anti-fibrotic actions. In this animal study, we demonstrated that steroid-eluting stents, through these dual effects, have therapeutic potential for BBS refractory to conventional methods. Clinical trials are required to confirm the safety and effectiveness of this type of stent in humans.

## Materials and methods

### Preparation of corticosteroid-eluting stents

**Figure 5:**
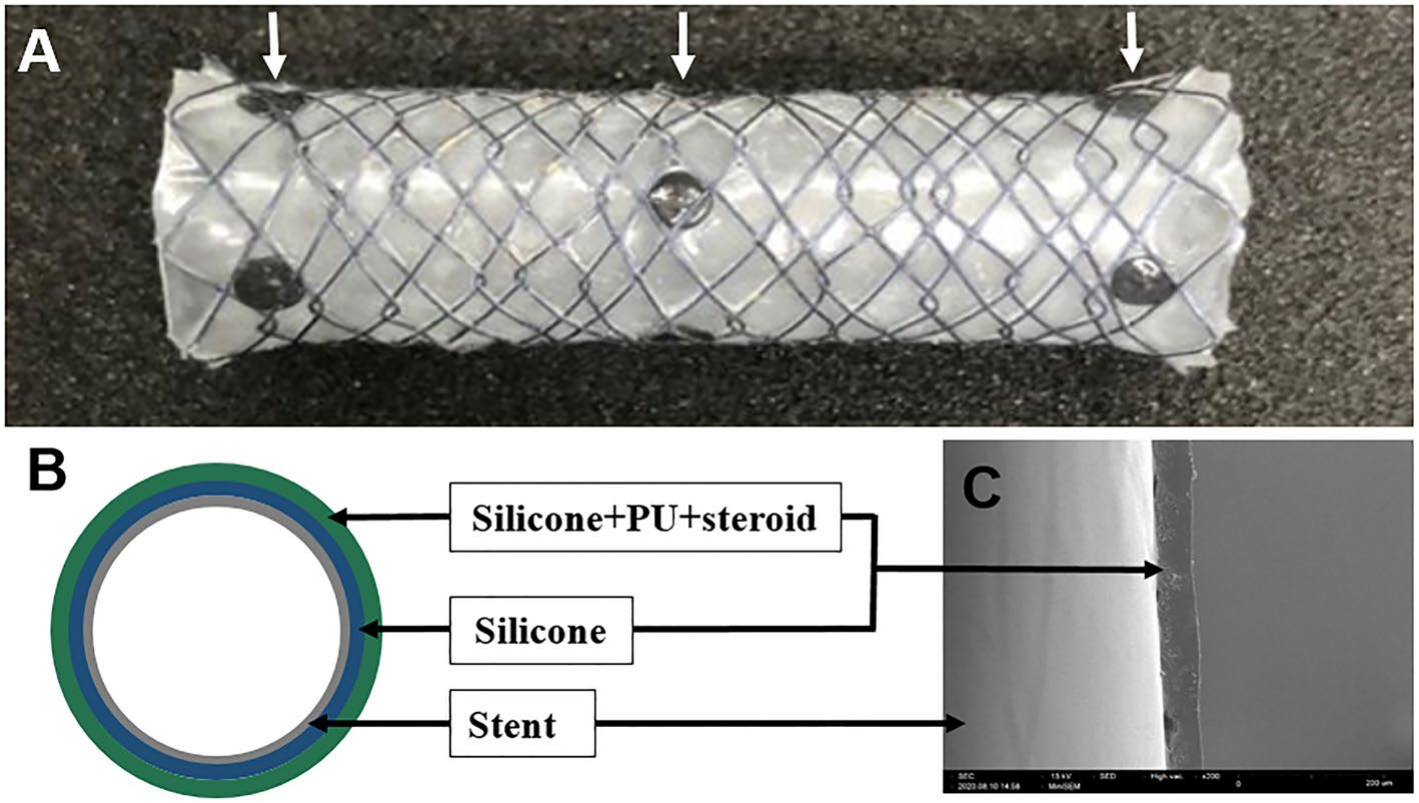
Fully covered self-expanding metal stent with a triamcinolone-incorporated membrane. (**A**) Photograph of the expanded stent with three radiopaque markers (in the middle and at both ends of the stent) used for proper positioning. (**B**) Schematic cross-section showing the silicone membrane forming the inner layer, and polyurethane membrane containing silicone and triamcinolone forming the outer layer. (**C**) Scanning electron micrograph of the stent membrane fabricated by the spray-coating technique, showing a uniform coating pattern. *PU* polyurethane. The thickness of the nitinol wire for the stent was 127 µm, and the hook and cross wire method was used for fabrication. Tantalum was used as the radiopaque marker material. Steroid refers to triamcinolone.

The stents were 6 mm wide and up to 20 mm long; the length was determined by the size of the lumen in which the stent was to be implanted. Each stent had three radiopaque markers for precise positioning at the stricture site (Fig. 5). The coating solution for the triamcinolone-eluting membrane comprised a primary coating solution, which was prepared by mixing silicon (MED-6640 A, B) and xylene; and a secondary solution prepared by dissolving the appropriate amount of triamcinolone (40 mg/mL) in a premixed solution of polyurethane and tetrahydrofuran (THF).

Coating was performed using the spray-coating technique. The concentration of corticosteroid in the membrane was controlled by adjusting the spray time. The area of the stent membrane was 25.12 cm^2^, and the corticosteroid concentration in the secondary solution was 300 µg/cm^2^. By controlling the spray time, the total amount of corticosteroid (triamcinolone acetonide; Sigma-Aldrich, St. Louis, MO, USA) in the stent membrane could be adjusted to 7.5, 15, or 30 mg in the hydrophilic polyurethane stents (Tecophilic™; aliphatic thermoplastic polyurethane [TPU]), and 15 or 30 mg in the hydrophobic polyurethane stents (Tecothane™; aromatic polyether-based TPU). We based these triamcinolone dosages on the existing literature. Previous studies of esophageal stents used 20–40 mg triamcinolone administered as one injection^15–17,22,34^. We therefore evaluated 15 mg, as well as 0.5- and twofold doses, to determine the optimal amount for the FCSEMS.

### In vitro drug release

To investigate the efficacy of drug release, the five types of triamcinolone-eluting membranes containing different types of polyurethane and amounts of triamcinolone were placed in phosphate-buffered saline for 6 days for in vitro drug release assay (three hydrophilic polyurethane groups [7.5, 15, and 30 mg triamcinolone] and two hydrophobic polyurethane groups [15 and 30 mg triamcinolone]).

### Formation of benign stricture and stent insertion

**Figure 6:**
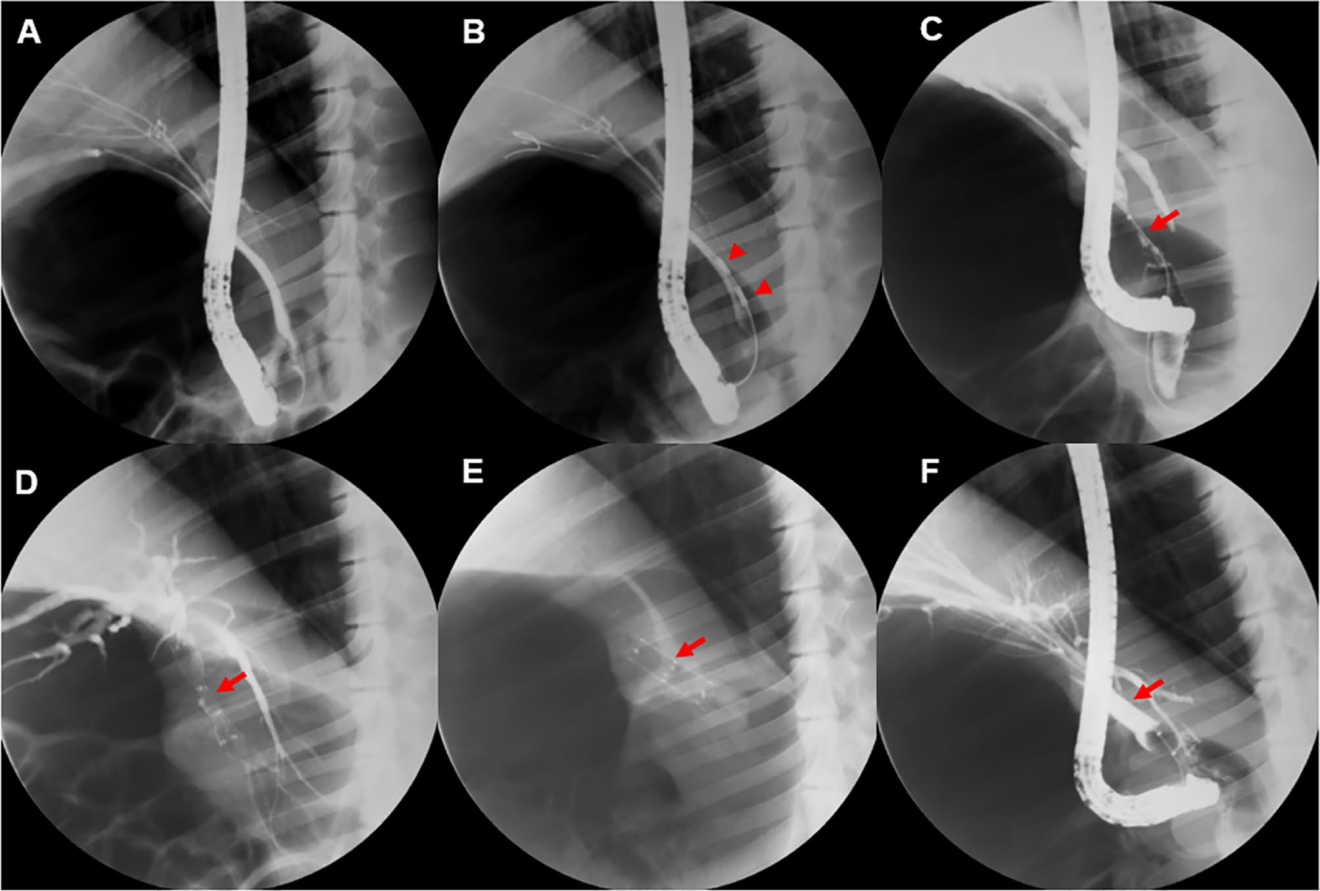
Experimental procedure. (**A**) Cholangiography showing no stenosis in a normal pig biliary tract. (**B**) Stricture model created in the mid-common bile duct using radiofrequency ablation (RFA). (**C**) At 4 weeks after RFA, the stricture was apparent, and the biliary tract was dilated proximal to the stricture. (**D**) The steroid-eluting stent was inserted endoscopically into the stricture. (**E**) The presence of stent migration was assessed by performing abdominal X-rays at 1-week intervals. (**F**) Cholangiography following stent removal at 4 weeks after initial insertion showing resolution of the stenosis. Red arrowheads indicate RFA lesions and red arrows show the stricture area.

In vivo experiments were performed using 12 female domesticated mini-pigs (*Sus scrofa*) with a mean weight of 27.9 kg (Supplementary Table 1). The animals were fasted for 12 h before the procedure, and drinking water was withheld on the morning of the procedure. General anesthesia was initiated by inhalation of 0.5–2% isoflurane with oxygen (1:1 ratio; 5–10 mL/kg/min) and endotracheal intubation was performed. The animals were placed on a fluoroscopy table in the left lateral position, and the endoscopic procedure was performed using an ERCP duodenoscope (TJF; Olympus, Tokyo, Japan). After selective cannulation of the common bile duct (CBD), an incision was made in the sphincter to facilitate insertion into the duct (Fig. 6).

Biliary stricture was established via intraductal RFA^33^. Twelve mini-pigs underwent ERCP-guided endobiliary-RFA (EB-RFA) using a temperature-sensing RFA catheter (ELRA RFA catheter®; STARmed, Goyang, Korea). After catheterization of the CBD, an initial cholangiogram was obtained, and a narrow segment of the duct was chosen as the site for EB-RFA. After positioning the RFA electrode close to the bile duct wall, radiofrequency energy was delivered using a VIVA combo radiofrequency generator (VCS10; STARmed) in target temperature-controlled mode (80 °C and 7–10 W for 120 s). In this mode, ablation is automatically terminated if the preset temperature is exceeded. Since purulent material was observed at 4 weeks after RFA in a previous study^33^, we inserted a 5 Fr single-pigtail catheter into the CBD to prevent cholangitis.

The 12 animals were divided into three groups (control: non-drug-eluting stent; steroid 1 × : stent with the optimal corticosteroid amount; and steroid 2 × : stent with 2 × the optimal corticosteroid amount), with four animals in each group (Supplementary Fig. 2). At 4 weeks after RFA, BBS of the CBD was confirmed by fluoroscopy, and control and experimental stents were then inserted into the stricture via ERCP under fluoroscopic guidance. All stages of this animal experiment have been approved by the Institutional Animal Care Committee of KNOTUS Co., Ltd., (KNOTUS IACUC: 20-KE-588). All methods were carried out in accordance with relevant guidelines and regulations. All methods are reported in accordance with ARRIVE guidelines.

### Follow-up after stent insertion

General symptoms, death, and body weight were recorded for each animal during the test period. Abdominal X-rays were taken every 2 weeks after inserting the stent to assess migration. To evaluate the development of cholestasis, we performed blood tests (complete blood count [CBC] and biochemistry) before RFA, 4 weeks after RFA, and 3, 14, and 28 days after stent insertion. At 4 weeks after RFA, ERCP was repeated to fluoroscopically measure the width and length of the stricture. Animals were euthanized at various times after stent insertion to obtain specimens for pathologic analysis.

### Extraction of specimens and histological analysis

An experienced pathologist (JHN) performed gross and histologic analyses of specimens obtained after stent insertion. After ERCP had been performed, the stent was removed, the CBD stenosis was photographed, and the diameter and length of the stenosis were measured. The entire bile duct, including the stricture site and tissues approximately 2 cm in diameter on both sides of the stricture bile duct, were removed and fixed in 10% formaldehyde. Paraffin sections of the entire bile duct were obtained and examined histologically after hematoxylin and eosin (H&E) and Masson trichrome staining. The slides were scanned to obtain digital images using a PANNORAMIC 250 scanner (3DHistech, Budapest, Hungary). The thickness of bile duct wall fibrosis (fibrous wall thickness) was measured. The thickest, thinnest, and average thickness was measured in a blue-stained region of dense collagen deposition under the muscle layer on Masson trichrome-stained slides (Supplementary Fig. 3). The distance measurement tool in CaseViewer software (3DHistech) was used to obtain these measurements.

### Primary and secondary outcomes

The primary outcome was the safety of steroid-eluting stents inserted into BBS created via temperature-controlled EB-RFA in normal swine CBDs. The secondary outcomes were the efficacy of steroid-eluting stents for treating these strictures and the optimal amount of corticosteroid within the stents.

### Statistical analysis

Quantitative data from the in vitro drug release and fibrosis wall thickness experiments are expressed as means ± standard deviation and were compared using Student’s t-test. Blood test results and experimental parameters are expressed as medians and range and were compared using the Mann–Whitney U test. All data were analyzed using SPSS software (ver. 12.0; SPSS Inc., Chicago, IL, USA). P-values < 0.05 were considered indicative of statistical significance.

## Supplementary Information

Supplementary Information.

## Data Availability

The datasets used and/or analysed during the current study available from the corresponding author on reasonable request. Figures were created using a window capture program (Window version 10.0).
