# Supplementary material for: Preclinical evaluation of endoscopic placement of a steroid-eluting metal stent in an in vivo porcine benign biliary stricture model
Source: Sci Rep. 2022 May 25;12:8864. doi: 10.1038/s41598-022-12957-0 (PMC9132970; doi:10.1038/s41598-022-12957-0)

**Supplementary Appendix**

This appendix has been provided by the authors to give readers additional information regarding their work.

Supplement to: Preclinical evaluation of endoscopic placement of steroid-eluting metal stent in an in vivo porcine benign biliary stricture model

Sung Ill Jang, Sungsoon Fang, Ji Hae Nahm, Jae Hee Cho, Min Young Do, Su Yeon Lee, Seok Jeong, Don Haeng Lee, Dong Ki Lee

**Contents**

**1. Supplementary Results: page**

**Supplementary table 1: page 2**

**Supplementary figure 1: page 3**

**Supplementary figure 2: page 4**

**Supplementary figure 3: page 5**

**1. Supplementary Results**

**Supplementary Table 1.** Characteristics of the eight female pigs that underwent insertion of a fully covered self-expanding metal stent with a triamcinolone-incorporated membrane.

| **Experimental group** | **Amount of triamcinolone (mg)** | **Animal number** | **Color** | **Weight (kg)** |
| --- | --- | --- | --- | --- |
| **Control** | 0 | A-1 | Black/yellow | 27 |
|  | 0 | A-2 | Black/white | 26.8 |
|  | 0 | A-3 | Black/yellow | 26.7 |
|  | 0 | A-4 | Black/white | 27.4 |
| **Steroid 1×** | 15 | B-1 | Black | 25.2 |
|  | 15 | B-2 | Black/yellow | 31 |
|  | 15 | B-3 | Black/yellow | 25.6 |
|  | 15 | B-4 | Black/white | 26.2 |
| **Steroid 2×** | 30 | C-1 | Black/white | 33 |
|  | 30 | C-2 | Black/yellow | 33.2 |
|  | 30 | C-3 | Black/yellow | 26.4 |
|  | 30 | C-4 | Black | 26 |

**Supplementary Figures**

**Supplementary Figure 1.** Body weight (A), WBC (B), RBC (C), Hb (D), PLT (E), AST (F), ALT (G), T-bil (H), and ALP (I) measured before radiofrequency ablation (RFA); 4 weeks after RFA; and 3 days, 2 weeks, and 4 weeks after stent insertion. In general, laboratory values increased after formation of the stenosis but showed a decreasing trend after stent insertion. No significant differences among groups were found at any time point. WBC, whole blood cell count; RBC, red blood cell count; Hb, hemoglobin; PLT, platelet count; AST, aspartate transaminase; ALT, alanine transaminase; T-bil; total bilirubin; ALP, alkaline phosphatase.


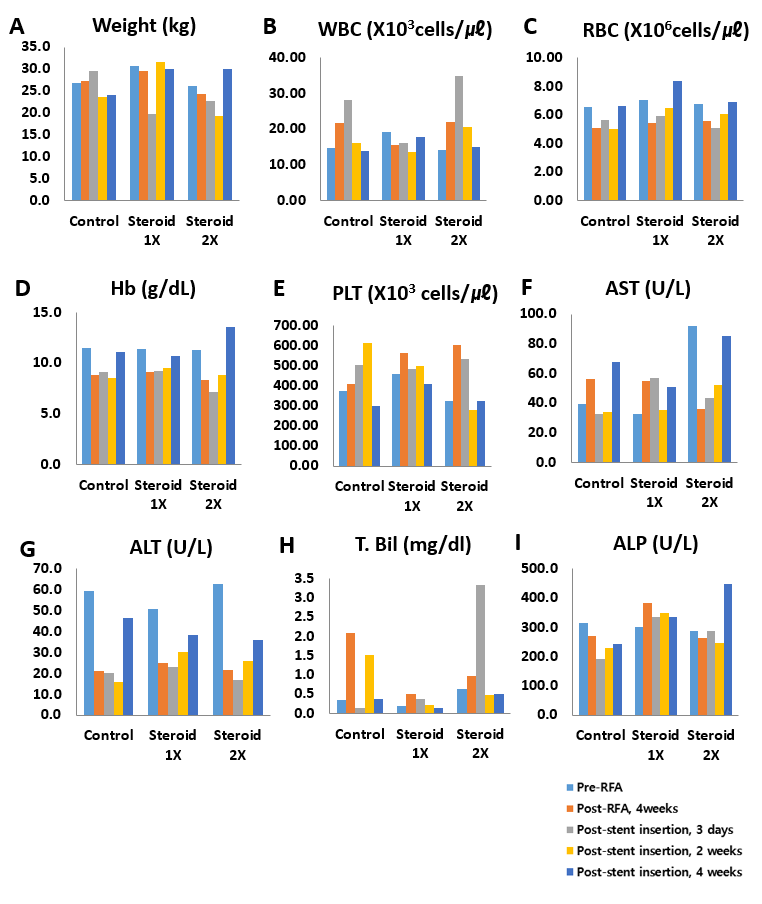


**Supplementary Figure 2. Experimental animal flowchart*.*** The group treatments were as follows: control, no steroid-eluting stent; steroid 1x, 15 mg triamcinolone steroid-eluting stent; and steroid 2x, 30 mg triamcinolone steroid-eluting stent. GI, gastrointestinal; RFA, radiofrequency ablation.

**
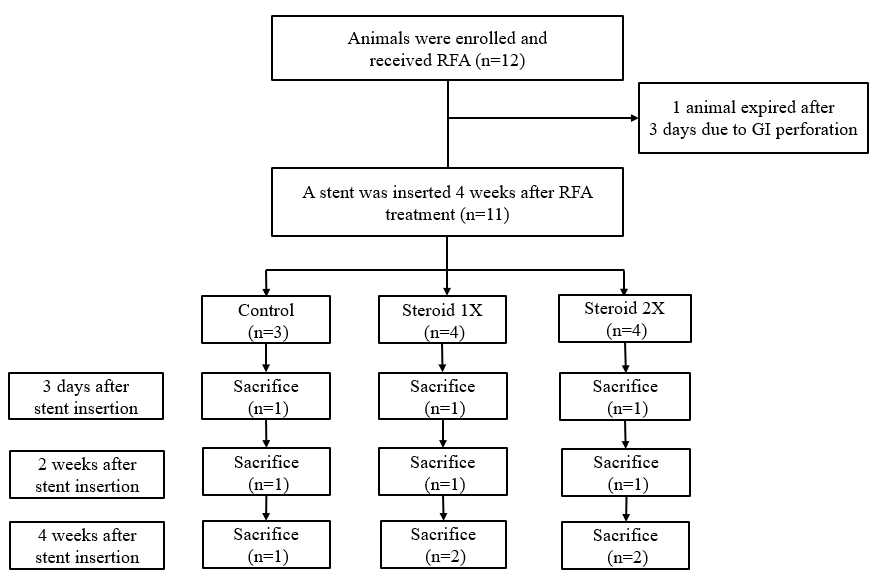
**

**Supplementary Figure 3**. Measurement of periductal fibrosis (fibrous wall thickness). The thickness of bile duct fibrosis was measured using the distance measurement tool in CaseViewer software (3DHistech). (A) The thickest, thinnest, and average thickness was measured in a region of dense collagen deposition under the muscle layer by Masson trichrome staining. (B) Blue line indicates magnification.


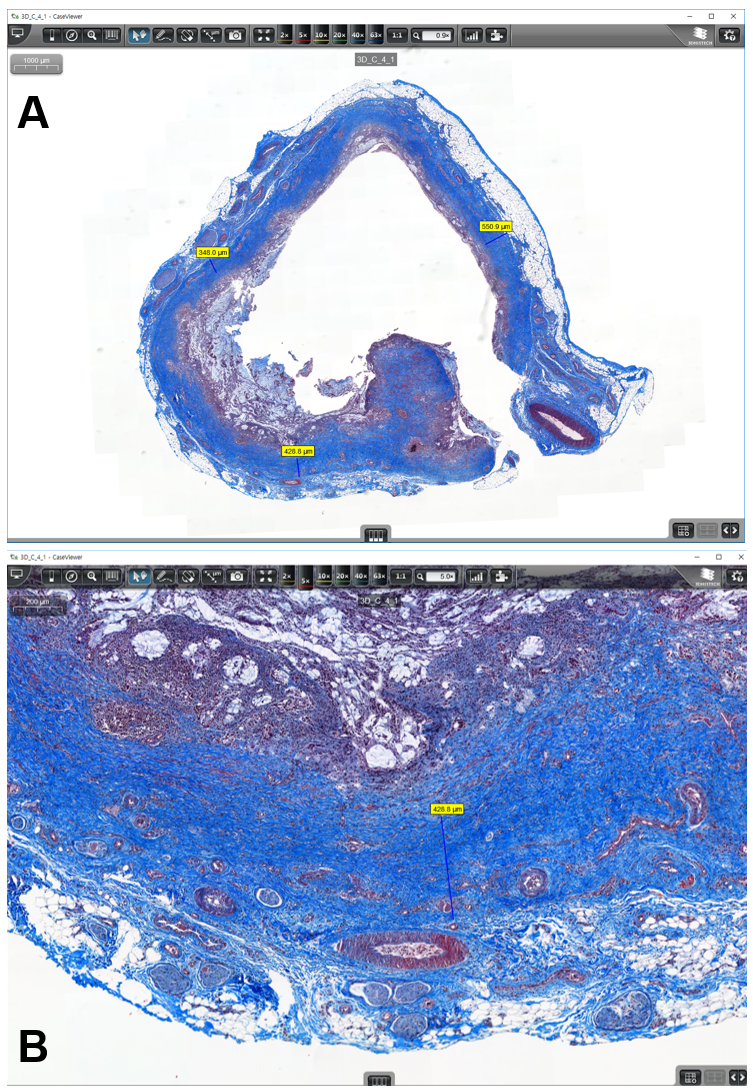

Supplement: Supplementary file 1 — Supplementary Information. [file 41598_2022_12957_MOESM1_ESM.docx]
